# International Technology Transfer of a GCLP-Compliant HIV-1 Neutralizing Antibody Assay for Human Clinical Trials

**DOI:** 10.1371/journal.pone.0030963

**Published:** 2012-01-27

**Authors:** Daniel A. Ozaki, Hongmei Gao, Christopher A. Todd, Kelli M. Greene, David C. Montefiori, Marcella Sarzotti-Kelsoe

**Affiliations:** 1 Department of Surgery, Duke University Medical Center, Durham, North Carolina, United States of America; 2 Department of Immunology, Duke University Medical Center, Durham, North Carolina, United States of America; University of Cape Town, South Africa

## Abstract

The Collaboration for AIDS Vaccine Discovery/Comprehensive Antibody – Vaccine Immune Monitoring Consortium (CAVD/CA-VIMC) assisted an international network of laboratories in transferring a validated assay used to judge HIV-1 vaccine immunogenicity in compliance with Good Clinical Laboratory Practice (GCLP) with the goal of adding quality to the conduct of endpoint assays for Human Immunodeficiency Virus I (HIV-1) vaccine human clinical trials. Eight Regional Laboratories in the international setting (Regional Laboratories), many located in regions where the HIV-1 epidemic is most prominent, were selected to implement the standardized, GCLP-compliant Neutralizing Antibody Assay for HIV-1 in TZM-bl Cells (TZM-bl NAb Assay). Each laboratory was required to undergo initial training and implementation of the immunologic assay on-site and then perform partial assay re-validation, competency testing, and undergo formal external audits for GCLP compliance. Furthermore, using a newly established external proficiency testing program for the TZM-bl NAb Assay has allowed the Regional Laboratories to assess the comparability of assay results at their site with the results of neutralizing antibody assays performed around the world. As a result, several of the CAVD/CA-VIMC Regional Laboratories are now in the process of conducting or planning to conduct the GCLP-compliant TZM-bl NAb Assay as an indicator of vaccine immunogenicity for ongoing human clinical trials.

## Introduction

The CAVD/CA-VIMC was established in 2006, in part, to create a global laboratory program for standardized assessments of antibody responses to viable vaccine candidates for HIV-1 [1]. This program's overall goals included expediting the development of an effective HIV vaccine through the contribution of validated assays, development of shared Standard Operating Procedures (SOPs), laboratory capacity building, and quality assurance oversight with adherence to GCLP guidelines for human clinical trials [2], [3]. These objectives were aligned with the Global HIV Vaccine Enterprise's Scientific Strategic Plans released in 2005 and 2010 [4], [5]. Toward this goal, the program placed an emphasis on engaging scientists at leading international institutions affiliated with potential international vaccine trial sites. Eight laboratories were selected to create an integrated network of Regional Laboratories, many representing regions where the HIV-1 epidemic is most prominent, to implement the standardized, GCLP-compliant conduct of the TZM-bl NAb Assay [6]. These laboratories were also selected due to their capacity to serve as regional training centers for further assay transfer within their country/region.

**Figure 1:**
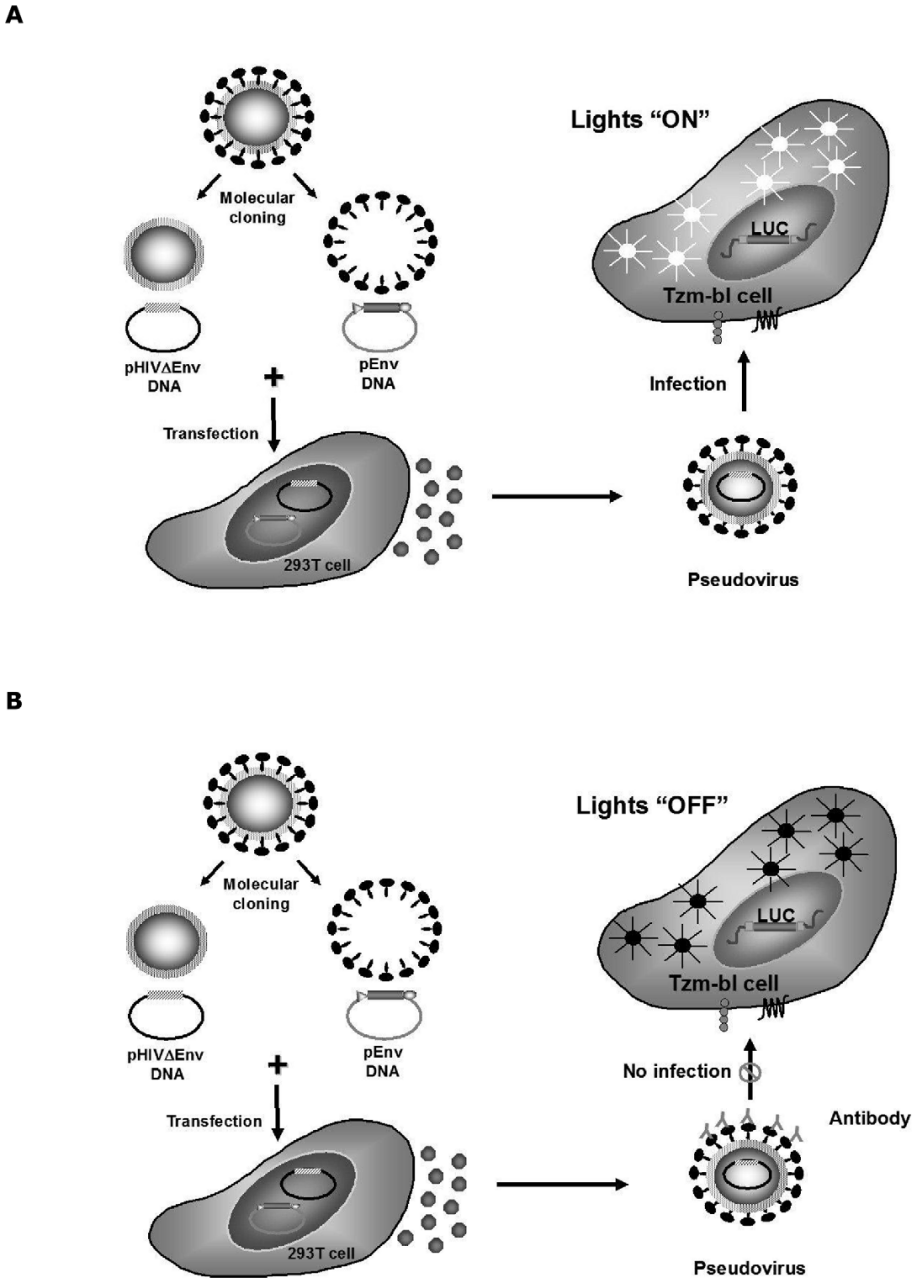
Pictorial representation of the TZM bl NAb Assay. Briefly, pseudovirus infection of TZM-bl Cells stimulates expression of Luciferase Reporter gene thereby emitting luminescence (A). When the pseudovirus is neutralized prior to the infection of TZM-bl Cells, the Luciferase reporter gene is not expressed and no luminescence is emitted (B).

The TZM-bl NAb Assay measures neutralization as a function of the reduction in Tat-induced luciferase (Luc) reporter gene expression after a single round of virus infection [6]. The TZM-bl cell, a HeLa cell clone engineered to express CD4 and CCR5 [7], [8], contains integrated reporter genes for firefly luciferase and *Escherichia coli* β-galactosidase which are under the control of an HIV-1 long terminal repeat (LTR) [8], thus permitting sensitive and accurate measurements of infection. TZM-bl cells are highly permissive to infection by most strains of HIV, including molecularly cloned Env-pseudotyped viruses (pseudoviruses). Pseudoviruses are created in 293T/17 cells by co-transfection with an Env-expressing plasmid and a backbone plasmid containing a defective Env gene. The co-transfected 293T/17 cells generate pseudovirus particles that are able to infect TZM-bl cells, but due to the absence of a complete genome, are generally unable to produce infectious progeny virions [6]. Expression of the luciferase reporter gene in TZM-bl cells is induced by the viral Tat protein following a single round of infection (see Figure 1) [6]. Luciferase activity is quantified by luminescence and is directly proportional to the number of infectious virus particles present in the initial inoculum [6].

The TZM-bl NAb Assay has several advantages over other neutralizing antibody assays (i.e. PBMC assay) [1], [6]. Use of a clonal cell line (TZM-bl) provides enhanced precision and uniformity [6]. Pseudoviruses offer advantages over uncloned virus including greater reagent stability and neutralization assay reproducibility [1], [6]. The assay was validated by the CAVD/CA-VIMC Central Reference Laboratory (CRL, directed by Dr. David Montefiori, Duke University Medical Center). The procedures associated with this validated assay have been developed into centrally controlled SOPs which are utilized by all CAVD/CA-VIMC laboratories conducting the TZM-bl NAb Assay.

In the past several years, this assay has gained recognition as one of the gold standard assays utilized for the measurement of the magnitude and breadth of HIV-1 vaccine-elicited neutralizing antibody responses [9]. As the data generated by testing HIV-1 vaccine-elicited neutralizing antibody responses could be potentially used in support of a licensing application to a regulatory authority, the laboratories were required by the CAVD/CA-VIMC to conduct all research under GCLP to ensure that results were reliable, repeatable, auditable, and comparable between multiple testing laboratories [2], [3], [10]. A Central Quality Assurance Unit (CQAU) was established for the consortium to implement the TZM-bl NAb Assay in a GCLP-compliant environment. The CQAU led an initial effort to harmonize existing GCLP guidelines for consistent management of laboratory operations in support of clinical trials [11]. These harmonized GCLPs were adopted for the studies described in this manuscript. To assure equivalent assay performance between the CRL and Regional Laboratories, the CAVD/CA-VIMC Operations Core and CQAU (CA-VIMC Core) developed an Assay Implementation Plan that outlined key experiments and procedures necessary to revalidate parameters of the assay in a GCLP-compliant environment. While literature describes the transfer of a T-cell based assay between laboratories in GCLP compliance [12], [13], this is the first effort, to our knowledge, of the transfer of neutralizing antibody assay technology in compliance to GCLP to multiple laboratories for use in global HIV vaccine clinical trials.

## Materials and Methods

### Ethics Statement

This study does not involve human subjects. This study utilized pre-existing, de-identified specimens and was conducted under the approval of the local Institutional Review Boards (IRBs). The following IRBs conducted oversight for their respective sites: Siriraj Ethics Committee (Bangkok, Thailand), Division of Human Subject Protection, Walter Reed Army Institute of Research (DHSP-WRAIR) (Bangkok, Thailand), Institutional Review Board for Chinese Center for Disease Control and Prevention/National Center for AIDS/STD Control and Prevention (Beijing, China), University of Witwatersrand – Human Research Ethics Committee (Human) (Johannesburg, South Africa), HIV/AIDS Research Committee of the Uganda National Council for Science and Technology (Kampala, Uganda), Medical Association of Saarland – Public Corporation Ethics Commission (Sulzbach, Germany), NARI Ethics Committee (Pune, India), and YRG CARE Institutional Review Board (Chennai, India). The data were analyzed anonymously.

### Participating Laboratories

As previously mentioned, the CAVD/CA-VIMC consisted of 8 Regional Laboratories (see Table 1). These laboratories were selected based on their experience conducting antibody assays for HIV vaccine research, their existing infrastructure to implement new assay technology, their capacity to serve as regional training centers for further assay transfer within their country/region, and their geographic proximity to the HIV epidemic and potential HIV vaccine clinical trials.

**Table 1:** CAVD/CA-VIMC Regional Laboratories that completed the Assay Implementation Plan.

Laboratory	Principal Investigator(s)	Location
Armed Forces Institute for Research Medical Sciences (AFRIMS)	Dr. Mark de Souza, Dr. Victoria Polonis	Bangkok, Thailand
Fraunhofer Institut für Biomedizinische Technik (IBMT)	Dr. Hagen von Briesen	Sulzbach, Germany
Makerere University – Walter Reed Project (MUWRP)	Dr. Fred Wabwire-Mangen, Dr. Victoria Polonis	Kampala, Uganda
National AIDS Research Institute	Dr. Ramesh Paranjape	Pune, India
National Center for AIDS/STD Prevention and Control (NCAIDS), China CDC	Dr. Yiming Shao	Beijing, China
National HIV Repository and Bioinformatic Center/Siriraj Hospital	Dr. Ruengpung Sutthent, Dr. Victoria Polonis	Bangkok, Thailand
National Institute for Communicable Diseases (NICD)	Dr. Lynn Morris	Johannesburg, South Africa
Y.R. Gaitonde Centre for AIDS Research and Education (YRG CARE)	Dr. Suniti Solomon, Dr. Pachamuthu Balakrishnan	Chennai, India

### TZM-bl NAb Assay

The TZM-bl NAb Assay described here is a modified version of the assay described previously [6], [14], [15]. Serologic reagents to be tested for neutralizing activity were serially diluted in 96-well flat-bottom culture plates containing Growth Medium, followed by the addition of Env-pseudotyped virus that was previously titrated for optimal infectivity. Freshly trypsinized TZM-bl cells, containing an optimal concentration of DEAE-Dextran (as determined in each laboratory), were added to each well following a 45–90 min incubation period. Following a 48 hr incubation period, culture medium was removed from each well and replaced with a luciferase reporter gene assay system reagent (Britelite, PerkinElmer or Brite-Glo, Promega). After a short incubation (minimum of 2 min), lysates were transferred to 96-well plates for measurement of luminescence in a luminometer. The 50% inhibitory dose (ID_50_) was defined as the reciprocal of the serologic reagent dilution that caused a 50% reduction in relative luminescence units (RLU) compared to virus control wells after subtraction of background RLU. Failure to score at least 50% reduction of RLU at any serum dilution constituted a negative test; this cut-off was established as being the midpoint in the linear portion of the neutralization curve (20–80% neutralization). This assay was formally validated for specificity (<2% false positive rate), precision (values within 3-fold for 80% of the determinations), linearity (r^2^>0.85), range (20–80% neutralization), lower limit of detection (+3.3 standard deviation (s.d.) of background), lower limit of quantitation (+10 s.d. of background), accuracy (within 95% confidence intervals), and robustness following ICH Q2R(1) [16].

### Pseudovirus Preparation

Pseudoviruses were manually prepared at Duke University Medical Center (Durham, NC USA) or at the Institut für Biomedizinische Technik (IBMT) (Sulzbach, Germany). 293T/17 cells (American Tissue Culture Collection) were seeded (3×10^6^–5×10^6^) in a T-75 cm^2^ tissue culture flask containing Growth Medium and incubated 20–24 hours at 37°C/5% CO_2_. The following day, transfection complexes were formed by combining an HIV-1 Env plasmid containing a Luciferase reporter gene and a backbone plasmid containing a defective Env-gene along with FuGENE 6 reagent (Promega, USA) and Dulbecco's Modified Eagle Medium (Gibco). The transfection complexes were allowed to incubate for 30 minutes at room temperature (18°–25°C). The complexes were then added to the flask of 293T/17 cells and incubated at 37°C/5% CO_2_ for 3–8 hours. Following a change of media after the 3–8 hour incubation, the cells were incubated at 37°C/5% CO_2_ for an additional 48–72 hours. The virus-containing media was then harvested from the flasks and filtered through a 0.45 µm filter, to eliminate cell debris. The concentration of FBS was brought up to 20% in the virus-containing medium and the medium aliquoted and frozen at −80°C.

### Tissue-Culture Infectious Dose Assay (Pseudovirus Titration)

Pseudovirus stocks were plated in quadruplicate and serially diluted in Growth Medium. Freshly trypsinized TZM-bl cells were added to the plate in GM containing an optimized concentration of DEAE-Dextran. The plate was incubated for 48 hours at 37°C/5% CO_2_. Following the incubation period, culture medium was removed from each well and replaced with a luciferase reporter gene assay system reagent (Britelite, PerkinElmer or Brite-Glo, Promega). After a short incubation (minimum of 2 min), lysates were transferred to 96-well plates for measurement of luminescence in a luminometer. The recommended virus dilution to use in the TZM-bl NAb Assay was calculated to ensure a standardized virus dose in the assays.

### Laboratory Assay Implementation Plan

The goal of the Implementation Plan was to ensure that all laboratories within the Consortium were performing the TZM-bl NAb Assay in a GCLP-compliant environment and were achieving comparable results. The Implementation Plan consisted of four Phases and each laboratory was required to successfully achieve pre-determined criteria in each Phase in order to complete the Plan. Phase I of the Implementation Plan focused on the initial training and transfer of the assay technology to each of the laboratories. Phase II outlined a series of procedures and experiments that aimed to implement and optimize the TZM-bl Nab Assay in the laboratories. Items such as cell maintenance and establishment of cell banks, equipment installation and validation, and determination of optimal concentrations for key reagents were addressed in this phase. During Phase II, the CQAU also conducted an initial site-visit to each laboratory and provided GCLP training to the laboratory staff [11]. The experiments detailed in Phase III of the Implementation Plan focused on the local revalidation of the assay through the analysis of robustness and precision. As a part of this phase, the laboratories were required to enroll in the formal Global Proficiency Testing Program for the TZM-bl NAb Assay administered by the CQAU [15]. The final phase, IV, consisted of a formal GCLP audit led by an auditor external to the CAVD/CA-VIMC CQAU with associated corrective actions and preventative actions (CAPA). The laboratories were also required to conduct trend analysis on control values and other key parameters that measure quality in the laboratory. For all phases, acceptance criteria were determined by the CA-VIMC Core based on the previous validation of the TZM-bl NAb Assay. The Plan was formalized into a CAVD/CA-VIMC Central SOP to ensure that each laboratory performed the identical set of experiments and followed the same procedures. The laboratories were required to submit the data from each Phase to the CA-VIMC Core for review and approval. Following the completion of all phases of the Implementation Plan, the CA-VIMC Core issued an Endorsement document to the laboratory stating that the laboratory could perform the TZM-bl NAb Assay in a GCLP-compliant manner for the CAVD. The Endorsement was valid for one year and was contingent upon the laboratory's successful completion of the semi-annual Global Proficiency Testing Program [15]. Formal GCLP audits were subsequently conducted annually for CAVD/CA-VIMC-funded Regional Laboratories and successful completion led to the re-endorsement of the laboratory by the CA-VIMC Core.

### Data Analysis

All ID_50_ values were calculated using a formally validated Excel-based macro or web-based Nab tool [17] that utilizes average virus and cell control RLU values as well as duplicate test well RLU values to calculate the neutralizing antibody titer as a function of the reduction of luciferase reporter gene expression. All means, standard deviations (s.d.), and r^2^ values were calculated using Microsoft Excel formulas. Percent coefficient of variation (%CV) was calculated by dividing the s.d. by the mean and multiplying by 100. For the initial on-site competency testing, values within 3-fold of the established truncated means (the highest and lowest ID_50_ values were excluded from the mean calculation for each serologic reagent/pseudovirus) were judged to be acceptable. The fold difference between the laboratory and the established mean was calculated by dividing the laboratory value by the mean value: values between 1/3-fold and 3-fold of the mean were considered acceptable.

## Results

### Initial Training

The CA-VIMC Operations and CRL developed an assay training program for visiting scientists. At least one key laboratory member from each laboratory was required to successfully complete this training at the CRL (one Regional Laboratory sent two individuals to complete training at a CAVD/CA-VIMC formally-endorsed Regional Laboratory, due to cost and time considerations). This training program followed a central assay training SOP which outlined a series of phases including the reading and understanding of assay SOPs, assay observation, assay performance with supervision, assay performance without supervision, and an initial competency test. The typical duration for the training program was four weeks and consisted of training on sterile technique, cell culture, pseudovirus preparation and titration (TCID assay), the TZM-bl NAb Assay, and data analysis. Each trainee was required to independently perform a competency test and meet pre-determined acceptance criteria prior to formal completion of the training program. Successful completion of the competency assessment required the trainee to achieve results within 3-fold of the reference values for at least 80% of serologic reagent/pseudovirus combinations.

### Assay Technology Transfer to Regional Laboratory

In preparation for implementing the assay on-site, the CAVD/CA-VIMC Operations partnered with each laboratory for the coordination, acquisition, and procurement of necessary assay reagents, cell lines, and equipment prior to, or during the assay training program. Following completion of the training program, the trainees were responsible for importing the Pseudovirus Titration and TZM-bl NAb Assay technologies to their laboratory and training the other laboratory personnel. The trainees were encouraged to practice performing the assay on-site with existing specimens in order to increase confidence in the procedure and techniques. The CA-VIMC Operations maintained consistent communication with each site to assist in reviewing assay data for acceptability and troubleshooting of any problems encountered.

### GCLP Compliance

The trainees were also encouraged to select areas of their laboratory that could be converted and dedicated to the conduct of GCLP compliant assays. Due to the relative novelty of GCLP principles at the onset of the Consortium, the CQAU worked with each site to introduce GCLP concepts and strategies and ensure that each laboratory was aware of the sponsor-driven requirements and expectations involved with GCLP implementation [2], [3], [10], [11]. In accordance with GCLP compliance, the CQAU asked each laboratory to designate an individual to serve as the site-specific Quality Assurance (QA) Coordinator. The QA Coordinator was responsible for implementing on-site CAVD/CA-VIMC Central SOPs directing the conduct of assay, equipment, management, training, data management, and reagent preparation procedures for the conduct of the TZM-bl NAb Assay in compliance to GCLP. To ensure that the QA Coordinator was knowledgeable of current GCLP guidelines, the CQAU encouraged QA Coordinators to attend formal GCLP training seminars [2], [3]. To ensure the delivery and receipt of Central SOPs, the CQAU utilized a Microsoft Sharepoint-based web portal for the secure distribution of Central SOPs to the QA Coordinator at each laboratory and devised a feedback mechanism whereby the QA Coordinator would confirm receipt of the SOPs via facsimile or email transmission to the CQAU. Additionally, the QA Coordinator was responsible for the local oversight of the quality program as it related to CAVD/CA-VIMC projects. Ideally, the QA Coordinator was independent from the conduct of any TZM-bl NAb Assays; however, in cases of limited laboratory capacity or budgetary constraints, laboratory personnel involved in the conduct of assays were allowed to serve as the QA Coordinator provided he or she did not conduct assays for the CAVD/CA-VIMC. The CQAU also assisted the laboratories with the development and refinement of site-specific SOPs that covered general equipment usage and maintenance, facility maintenance, reagent acquisition, labeling, and maintenance, quality assurance systems, management processes, specimen management and transportation, and data management and storage procedures. Additionally, the CQAU assisted the laboratories in assembling personnel/training records, disaster recovery plans, quality management plans, reagent inventories, auditing processes, annual competency assessments, and change control processes, all in support of the establishment of GCLP compliance. The CQAU provided templates and examples of the documents to the laboratories as reference in developing their own procedures.

### TZM-bl Cell Culture and Maintenance

The laboratories were required to create both Master Archive and Master Working Stocks of cryopreserved cells in order to preserve the integrity of the cell line. In order to assess the ability to properly maintain cell lines in culture without contamination, the laboratories were required to perform quality control testing for *Mycoplasma* contamination at weeks 0, 2, 4, 8, 12, 18, and 24 of culture, yielding negative test results for the entire duration to assure the quality of the stocks. Each Regional Laboratory conducted testing via on-site utilization of a commercially available *Mycoplasma* detection kit or submission of cells to a third party *Mycoplasma* testing facility. The results were required to be submitted to the CA-VIMC Core for review and approval. Based on the data submitted, none of the laboratories experienced *Mycoplasma* contamination of the Master Archive Stocks or the Master Working Stocks.

### Luminometer Installation and Qualification

**Figure 2:**
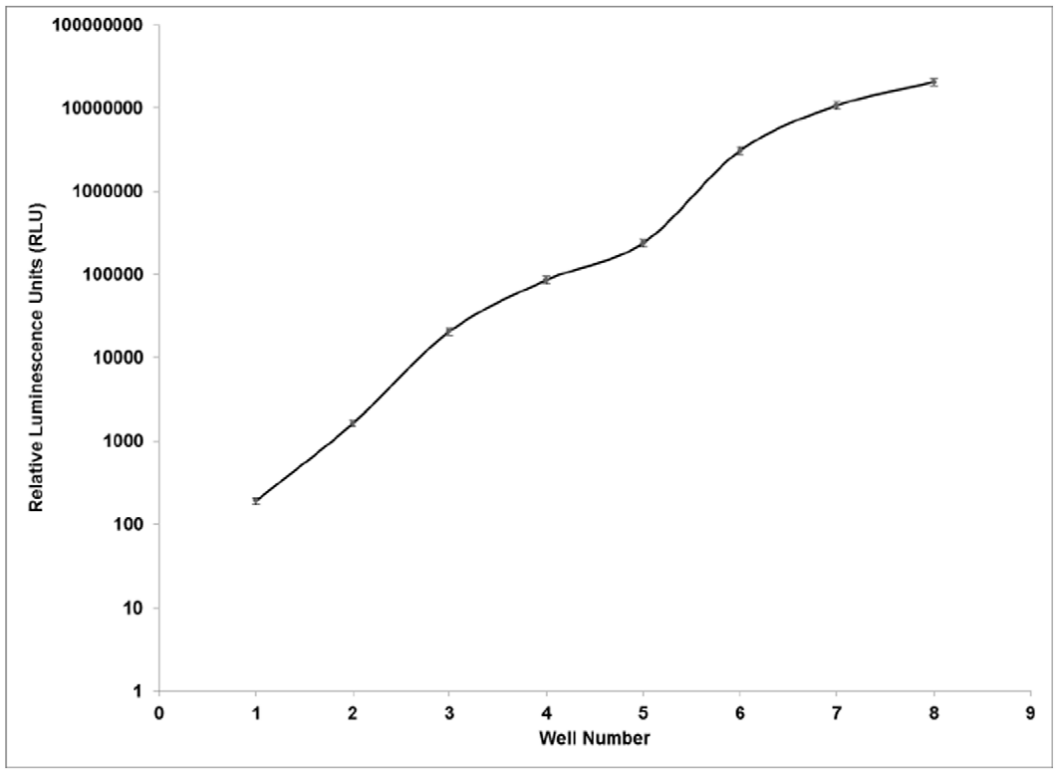
Example of a luminometer validation curve. Each month the values of a NIST-traceable-calibrated validation plate are plotted on a graph. The graphs are then superimposed and the values must remain within 10% of the established baseline (mean of the 20 initial runs).

The proper installation and validation of the luminometer was critical as it is a key piece of assay equipment. The initial installation, operation, and performance qualifications were performed by a service representative from the purchasing company. In order to monitor the performance of the instrument over time, the CQAU purchased and distributed a National Institute of Standards and Technology (NIST)-traceable Luminometer Reference Microplate to each laboratory from Harta Instruments, Inc. The plate utilizes a lithium ion battery and consists of a series of eight wells, each emitting varying levels of luminescence. The luminometer performance baseline reading was established by averaging the RLU values for each well over 20 individual plate runs in each laboratory. The performance of the luminometer was assessed on a monthly basis through comparison of monthly runs to the baseline readings in order to analyze the precision of the instrument (see Figure 2). Each reading had to be within 10% of the established mean value for the well in order to pass. Readings outside of 10% of the established mean value were required to be addressed with documentation and corrective action which included luminometer service/re-calibration. Regional Laboratories were instructed to re-establish the baseline readings following annual calibration of the reference plate.

### Determination of the Optimal Concentration of DEAE-Dextran

**Figure 3:**
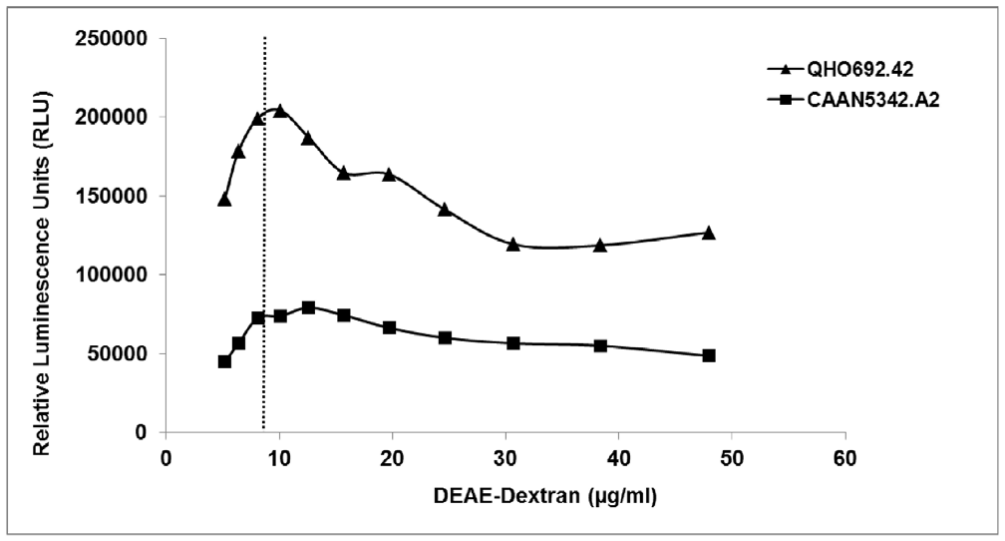
Example of a DEAE-Dextran titration curve using two pseudoviruses. The optimal concentration of DEAE-Dextran (x-axis) to use in the TZM-bl NAb Assay is calculated by selecting a concentration lower than the concentration yielding the peak RLU values on both titration curves of two pseudoviruses (in this instance QHO692.42, CAAN5342.A2). By picking the concentration lower than the peak, one avoids potential cell toxicity that may result with the use of other pseudoviruses. The vertical dotted line in the graph represents the concentration of DEAE-Dextran that maximizes the infectivity of the pseudovirus without being toxic to the TZM-bl cells.

DEAE-Dextran is a polycationic reagent that enhances the infectivity of the pseudoviruses [6]. The polycation counters the repulsive electrostatic forces between the virus and cells surface without affecting antibody binding and neutralization. However, DEAE-Dextran from different sources and different lots may exhibit substantial variability in potency and cell toxicity. This reagent can be toxic to TZM-bl cells at too high of a concentration. Therefore, it is imperative that the reagent be titrated to determine an optimal concentration for use in the assay. After acquisition of each lot of DEAE-Dextran by the laboratory, a stock was prepared and titrated using two pseudoviruses following a Central SOP. The laboratories were required to demonstrate that the optimal concentration that was selected for use in the laboratory did not exhibit toxic effects to the TZM-bl cells (see Figure 3).

### Pseudovirus Titration

**Figure 4:**
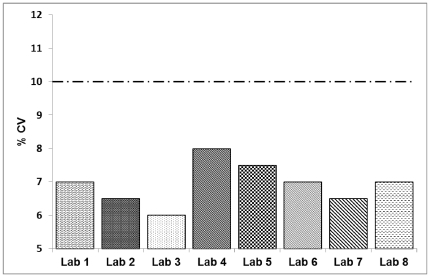
Average %CV of the replicate wells for the virus titrations that achieved “pass” criteria. The %CV of replicate wells was required to be lower than 10% at least 80% of the time.

Pseudoviruses used in the Assay Implementation Plan were created either by the CRL or by the CAVD/HIV Specimen Cryorepository (HSC) at the Fraunhofer IBMT (Sulzbach, Germany). Following preparation and prior to distribution, each pseudovirus was titrated and assigned a recommended dilution for use in the TZM-bl NAb Assay; however, subsequent tests have shown that the optimal dilution of virus to use may be laboratory-dependent due to differences in cell stocks, reagent vendors, etc. In order to examine the laboratory's ability to titrate the virus and calculate the correct virus dose for use in the TZM-bl NAb Assay, the laboratory was required to titrate two different pseudoviruses in TZM-bl cells using the TCID Assay. In order to pass this stage of Phase II, the laboratory was required to calculate the correct virus dose for use in the TZM-bl NAb Assay by determining the corresponding virus dilution based on the luminescence data from the TCID assay. The selected dilution should not have shown any evidence of cell killing in the assay. Additionally, to address precision and intra-assay repeatability, the replicate wells in the TCID assay were required to have a %CV of less than 10% for at least 80% of the replicate wells (pre-set criteria). It took laboratories an average of 1.4 attempts (s.d. 0.6) to pass the criteria pre-set in the Plan. Data for the average %CV between the replicate wells of pseudovirus titrations that passed are shown for each laboratory in Figure 4.

### Neutralization Assays

**Figure 5:**
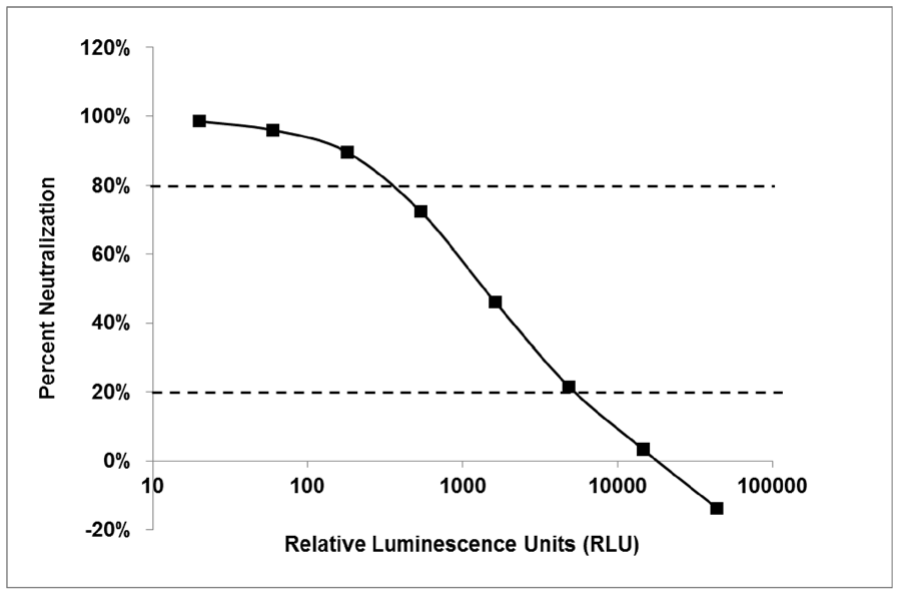
Example of Neutralization Curve. The neutralization curve depicts the linear portion of the curve between 20% and 80% neutralization.

Neutralization assays were conducted using the optimal concentration of DEAE-Dextran and standard dose of pseudoviruses derived from the previous experiments. The laboratory was required to assay five serologic reagents against two different pseudoviruses. The following pre-defined acceptance criteria (listed in the Central SOP) were used to judge the quality of the assays: 1) average RLU for virus control wells is greater than 10 times the average RLU for the cell control wells; 2) the %CV of the virus control wells is ≤30%; 3) the %CV of the sample wells is ≤30% for sample dilutions yielding at least 40% neutralization; 5) the neutralization curve is sigmoidal and approximately linear around 50% (example shown in Figure 5); and 6) the TZM-bl cells look healthy and are not subjected to analyte toxicity or virus-induced cell killing. As an additional measure for acceptability, the r^2^ value for the linear portion of the neutralization curve was calculated. Based on the validation data, the r^2^ value of the linear portion of the neutralization curve between 20% and 80% should be ≥0.85. While there were instances (11 out of 80) in which neutralization curves did not reach 80% (4 with no neutralization, 7 with partial neutralization under 80%), those that showed partial neutralization (<80%) had r^2^ values for their linear portions that were >0.85. All laboratories successfully passed this stage of the Plan.

### Initial Site-Visit by CQAU

The CQAU conducted an initial site-visit to each of the Regional Laboratories to examine the level of pre-existing compliance to GCLP and laboratory set-up. Each visit lasted 2–3 days and included the examination of items in the laboratory such as: facilities and equipment, personnel, specimen management, reagent acquisition, labeling, and maintenance, operator training and competency, quality assurance program, data management and information technology (IT), and archives. The CQAU also conducted GCLP training at the sites in order to provide an introduction to GCLP guidelines to the laboratory staff [2], [3], [10], [11]. Additionally, the CQAU discussed expectations of the laboratory with the local QA Coordinator and answered queries regarding the project. The CQAU composed a report of the laboratory visit that included suggestions for corrective actions to implement in order to better comply with GCLP.

### Initial On-site Competency Evaluation

**Figure 6:**
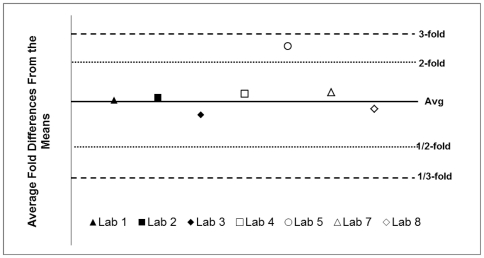
Average fold difference between laboratory values and means during initial on-site competency testing. Laboratories were required to achieve a relative ID_50_ value within 3-fold of the mean ID_50_ value (established from CRL and all other participating laboratories) for a minimum of 80% of reagent/pseudovirus combinations. Only 7 of the laboratories used the identical batch of competency test kits. The other laboratory used a similar kit from the Proficiency Testing program and was able to achieve the pre-determined pass/fail criteria for the program (data not shown).

The CA-VIMC Operations developed and implemented a competency testing program for the TZM-bl NAb Assay to ensure that the assay was being performed properly at each laboratory. Following a study plan, the CRL prepared and assembled competency test kits containing 10 blinded serologic reagents and 5 pseudoviruses. Each laboratory received the study plan, testing instructions, and a competency test kit to be performed on-site by the laboratory technician who received training at the CRL. The laboratories completed the competency evaluation and submitted their results to the CA-VIMC Core using a standardized data report. Competency test reference ID_50_ values were established by measuring the truncated mean for all data points. Neutralizing antibody ID_50_ values measuring within 3-fold of the truncated mean ID_50_ values, for at least 80% of the serologic reagent/pseudovirus combinations, were deemed acceptable. The average percentage correct (value for serologic reagent/pseudovirus combination within 3-fold of established mean) was 95.4% (s.d. 7.4)(n = 7; One laboratory was allowed to use the proficiency testing kit to serve also as their competency evaluation due to time constraints; therefore, their values were judged against the gold-standard reference values of the Proficiency Testing Program [15]). Individual performance reports were distributed to each laboratory noting the test results and suggesting areas of improvement based on examination of the raw data. As a further measure of how close the data were to the established truncated means, each laboratory's experimental ID_50_ values were compared to the corresponding mean ID_50_ values and a “fold-difference” was calculated by dividing the experimental value by the mean. The average of the “fold-differences” was then calculated and is graphed in Figure 6. The objective of using the average “fold-difference” is to determine how close a laboratory's values were to the mean values across all serologic reagent/pseudovirus combinations.

### Robustness Experiments

Robustness experiments were conducted by each laboratory to determine the effect of varying either the optimal virus dose or the optimal cell concentration for use in the TZM-bl NAb Assay. Previous validation data suggests that varying the input virus dose over a 100-fold range has little effect on assay sensitivity, i.e., the ID_50_ values of each serologic reagent differed by <3-fold (data not shown). The laboratory chose one previously titrated pseudovirus to conduct five parallel experiments examining the effect of pseudovirus dose on the neutralization by five serologic reagents. Experiments utilized the optimal dose of pseudovirus, as derived above, three times the optimal dose, ten times the optimal dose, one-third of the optimal dose, and one-tenth of the optimal dose. Results were submitted to the CA-VIMC Core for verification. Passing criteria indicated that positive neutralization curves should be approximately linear between 20% and 80% neutralization. As stated above, for curves that did not reach 80% neutralization, the CA-VIMC Core deemed the data acceptable using the criteria stated in the Central SOP.

**Figure 7:**
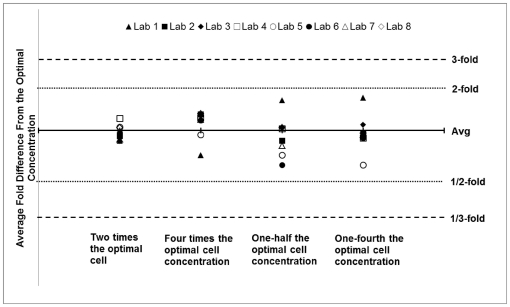
Results from robustness experiments. Average fold difference between ID_50_ values calculated from robustness experiments that varied the number of cells that were used in the assay. Assays were performed using the optimal cell number, twice the optimal cell number, four times the optimal cell number, one-half of the optimal cell number, and one-quarter of the optimal cell number. The ID_50_ values for each condition were required to remain within 3-fold of the ID_50_ values generated by the optimal cell number.

To analyze the effect of varying TZM-bl cell concentration, five parallel experiments were conducted utilizing one previously titrated pseudovirus assayed against 5 serologic reagents. The experiments were identical except for the amount of TZM-bl cells that were used. The laboratory examined the effect of using twice the optimal number, four times the optimal number, one-half of the optimal number, and one-fourth of the optimal number of cells and compared that to the neutralization ID_50_ values generated in the assay using the optimal number of cells. The laboratory was required to examine the quality of the cells via microscopic examination and the results of the experiment from the optimal cell concentration experiment had to be within 3-fold of the results derived in the neutralization assays described in Phase II. As a way to assess the effect of increasing or decreasing the number of cells that are placed into the assay, each laboratory's ID_50_ values were calculated using the optimal number of cells and compared to each of the other ID_50_ values that were calculated based on the conditions listed above. As seen in the graph, the larger the deviation from the optimal cell number, the more variation exists between calculated ID_50_ values (see Figure 7).

### Specificity

The presence of false positives in the TZM-bl NAb Assay could confound analyses performed to judge the potential efficacy of vaccine candidates. Thus, the laboratories were required to calculate the rate of false positives using the TZM-bl NAb Assay in their laboratory. The laboratory was required to assay 10 known HIV-1 seronegative serum samples against a well characterized pseudovirus (SF162.LS). The false positive rate established by the laboratory was required to be less than 10% in order to pass. All laboratories demonstrated a false positive rate of less than or equal to 10% (data not shown).

### Precision

**Figure 8:**
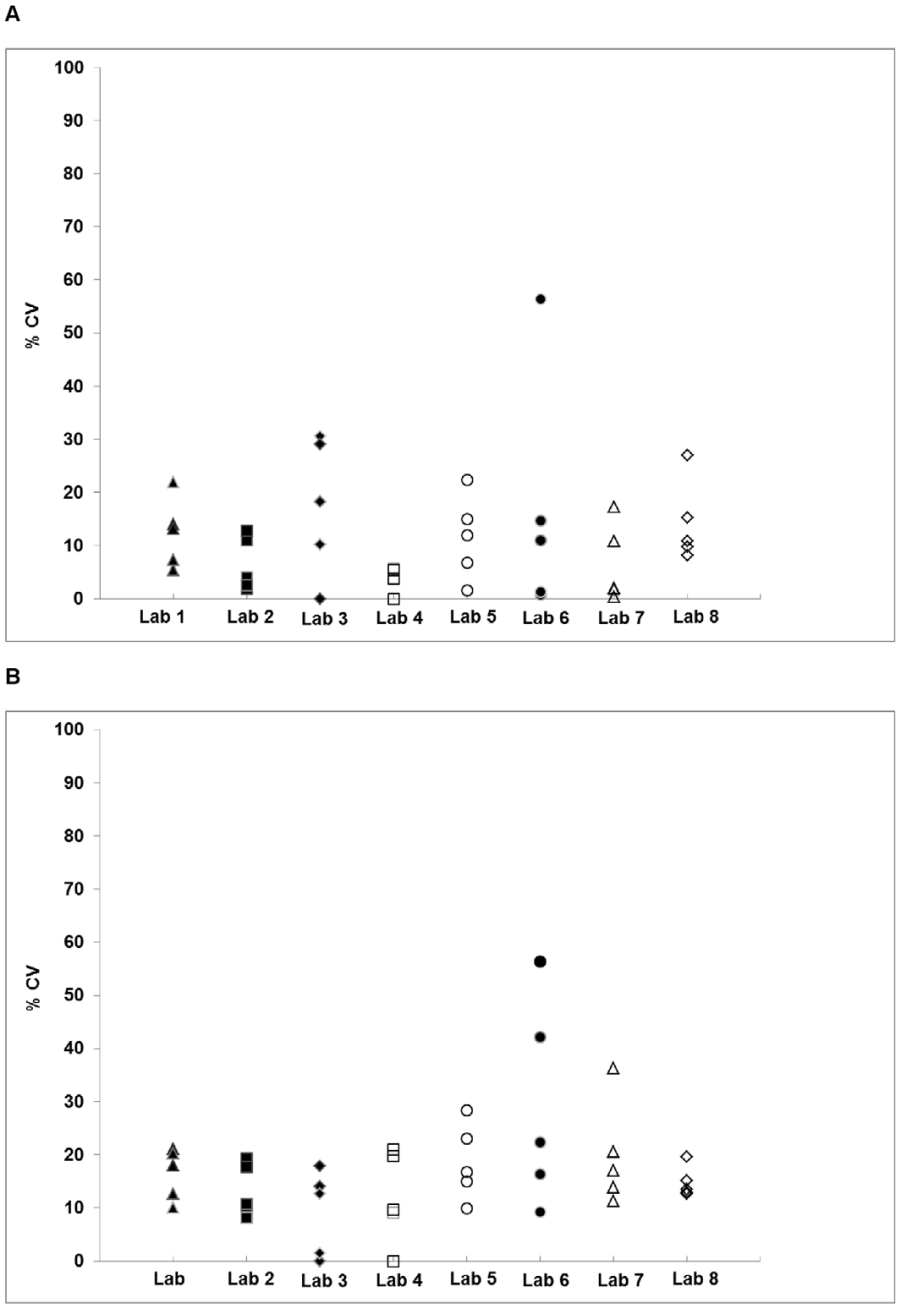
Results from precision experiments: inter-operator and inter-assay. (A) Results show the %CV between the ID_50_ values generated by two operators carrying out the identical experiment on the same days. Each point represents one sample/pseudovirus combination. (B) Results show the %CV between ID_50_ values generated from identical experiments conducted by the same operator on 3 different days. Each point represents one sample/pseudovirus combination.

Intermediate precision is defined by the variations within laboratory; specifically, between operators, assays, and assay dates [16]. Each laboratory was required to assess inter-operator variability (identical assays run by two different operators on the same day), inter-assay variability and intra-operator variability (identical assays run by the same operator at different time points). The calculated ID_50_ values between assays and/or operators were required to be within 3-fold at least 80% of the time. Figure 8 shows the average %CV between the ID_50_ values generated in the inter-operator experiments (top) and inter-assay experiments (bottom).

### Proficiency Testing Program

The first Standardized Proficiency Testing Program for the TZM-bl NAb Assay was pioneered by Duke University Medical Center in 2005 and formally implemented in 2009 through collaborations with National Institutes of Health/Division of AIDS (NIH/DAIDS) and the CAVD. The program enabled the comparison of each laboratory's results using identical test kits [15]. Each CAVD/CA-VIMC Regional Laboratory is required to enroll in the program and successfully complete assessments every 6 months. Furthermore, the CQAU mandates that the laboratory rotate the technician performing the proficiency testing to provide a more accurate assessment of the entire laboratory's performance over time. All Regional Laboratories have successfully achieved a passing score of at least 83% to date.

### Formal GCLP Audit

As a measure of the laboratory's compliance to GCLP, the CQAU conducted a formal audit of each laboratory with the presence of an external auditor to eliminate bias. The audit was conducted using a Master Audit Plan and Checklist that was developed as a result of harmonization efforts of GCLP guidelines [11]. The audit covered topics such as facility, equipment, organization, personnel, SOPs, quality program, data handling and integrity, IT, reagent acquisition, reagent labeling, reagent maintenance, and archives. Additionally an in-process audit of the TZM-bl NAb Assay was conducted to ensure that the practices were compliant with the Central SOP. Following the audit, the laboratory was given the audit report along with an audit report response form to which it was required to provide corrective action responses to findings of the audit within 20 business days. The CQAU then reviewed the responses from the laboratory and followed up with any additional items to be addressed. The rectification of common findings that were reported at the initial site-visit (lack of a functional Quality Management Plan, reagent bridging procedure, document archival process/facility, Disaster Recovery Plan, and processes for ensuring the integrity of IT systems) was verified by the CQAU before closing the formal audit process.

### Trend Analysis and Metrics

As an ongoing activity for each laboratory, analysis and trending of certain assay-related quality indicators was required. Each laboratory established positive controls for the TZM-bl NAb Assay. Fluctuating positive control ID_50_ values could signal potential variation within the assay and would result in the need for troubleshooting. The laboratories were required to monitor positive control data generated from each experiment over time for adherence to pre-established criteria. Other analyses such as the trending of average cell and virus control well values were also recommended to the laboratories. During the annual audit, the CQAU reviews the trending of quality indicators, which are monitored by the local QA Coordinator, and are performed by the site throughout the year under standard workload conditions.

### Endorsement

Following successful completion of all Phases of the Implementation Plan, the CA-VIMC Core provided a report to each of the laboratories that summarized the analysis of the submitted Implementation Plan data. Upon the successful completion of the audit and ensuing audit issues/responses, an Endorsement was issued to each of the laboratories that stated the laboratory's ability to conduct the TZM-bl NAb Assay in a GCLP-compliant environment for the CAVD. The Endorsement was valid for one year and was issued under the contingency that the laboratory continue to subscribe to and successfully complete the Proficiency Test as administered by the TZM-bl Standardized Proficiency Testing Program and also that the laboratory successfully complete an annual audit by the CQAU.

### Average Time for Implementation Plan Completion

All Regional Laboratories successfully completed the steps outlined in the Implementation Plan. On average, the time required for the laboratories to complete all of the phases was 12.8 months (s.d. 9.4). The shortest amount of time required for completion of the Plan was 6 months.

## Discussion

The assays and requirements outlined above represent critical aspects of the transfer of the TZM-bl NAb Assay to Regional Laboratories in a GCLP-compliant environment. This Program demonstrated that successful technology transfer was attainable across physical and cultural barriers in the global setting. It was imperative that the laboratories involved in the technology transfer perform revalidation experiments to document and prove that the locally-imported assay performed equivalently to the original validated assay. This assay has become widely used for the evaluation of the immunogenicity of candidate vaccines in Phase I/II clinical trials.

All laboratories successfully completed the Implementation Plan and were formally endorsed by the CA-VIMC to perform the TZM-bl NAb Assay for human clinical trials on behalf of the CAVD. However, there were some challenges that were encountered while working with laboratories in the international setting with respect to the initial technology transfer and completion of the Implementation Plan. The CA-VIMC program originally planned for the training of one key laboratory member at the CRL at program initiation. However, there were unanticipated delays in training program participation due to the lengthy process (e.g. average 8 months) associated with obtaining the required US visa authorization. During this interim period, the laboratories planned to obtain key reagents and equipment such that the assay implementation could begin immediately following the extensive training at the CRL. However, some sites faced challenges in obtaining local government approval for the utilization of funding as well as the importation of key reagents and equipment. For this reason, many of the trainees could not immediately begin assay implementation at their respective sites. Moreover, these Regional Laboratories were originally selected based on their extensive HIV vaccine research experience and affiliation with potential international vaccine trial sites. However, some of these laboratories were relatively inexperienced in the study of neutralizing antibodies in GCLP compliance, which posed initial challenges with assay design and quality control as well as data interpretation. In response to these challenges, the CA-VIMC Operations provided individualized technical support and oversight through close monitoring and frequent communication. Additionally, the CRL provided assay training to additional members of Regional Laboratories that requested further assistance; and, in some instances the CA-VIMC Operations provided training on-site. As far as general laboratory compliance to GCLP, there were several common findings among the laboratories that were identified during the initial site-visit by the CQAU. Lack of a functional Quality Management Plan, reagent bridging procedure, archival facility/process, Disaster Recovery Plan, and procedures for ensuring IT integrity were noted at a majority of the sites. The CQAU worked with each laboratory by defining expectations and also providing templates of documents that could be used to satisfy the requirements. All deficiencies identified at the initial site visit were corrected and verified by the CQAU prior to endorsing the laboratory.

In addition to these logistical and technical issues, language barriers were a challenge in several of the countries. The CQAU had the centrally-distributed SOPs professionally translated into several different languages. Furthermore, it was necessary to translate site-specific SOPs from their native languages into English to assure these processes and documentation were adherent to GCLP guidelines. Similarly, the CA-VIMC Core realized that formats for recording dates were different among the Regional Laboratories. To eliminate confusion between date-recording systems, the CQAU required all dates recorded for Consortium-related work to be notated, “dd/MMM/yy”. Additionally, one site followed the Buddhist calendar, where recorded dates are exactly 543 years ahead of the Gregorian calendar. Thus it was decided that all laboratories must record year dates according to the Gregorian calendar for consistency.

There were also challenges with meeting some of the acceptance criteria stated in the Implementation Plan which were adopted from the original validation of the TZM-bl NAb Assay. The validation demonstrated that the neutralization curves obtained for potent serologic reagents against well-established pseudoviruses reached 80% neutralization. Since the exact serologic reagent/pseudovirus combination was not specified by the CA-VIMC Core to the Regional Laboratory for use in conducting the neutralization assays, the combinations that were selected did not always reach 80% neutralization. Failure to achieve 80% neutralization is not necessarily indicative of a failed assay or poor assay technique as there are some serologic reagents that do not generate 80% neutralization against the selected viruses. Thus, in hindsight, the analysis of neutralization curves for linearity between 20–80% is not an effective indicator of assay performance. Additionally, a few laboratories had difficulty selecting the optimal concentration of DEAE-Dextran for use in the assay. While the concentration most often selected by the laboratories was the one that yielded the highest RLU for a particular pseudovirus, the laboratories were instructed to select a slightly lower concentration to avoid possible toxicity to the cells. Although laboratories were trained on how to properly select the optimal concentration, the CA-VIMC Core attributed this common issue to the quality of the SOP. To remedy this situation, the SOP was revised and the CA-VIMC Core provided technical support, reviewed each laboratory's data, provided multiple examples, and solicited laboratory feedback to ensure the laboratories knew how to properly select an optimal concentration. Finally, the laboratories had problems with the conduct of the pseudovirus titration assay. The four replicates at each dilution had to be within 10 %CV at least 80% of the time. Specifically, there was greater variation in the RLU values among the four replicates at very high and very low virus dilutions in the titration plate. Low dilutions (i.e. 1∶10, 1∶50) yielded very high and variable RLU values; thus raising the %CV. On the other end, at very high dilutions (i.e. 1∶3,906,250, 1∶19,531,250), the RLUs are very low and very minor changes could still have a large effect on the %CV. While many of the laboratories had to repeat particular pseudovirus titrations, they all were eventually able to pass the pre-set criteria of 10 %CV at least 80% of the time.

As a result of the efforts to standardize the conduct of the TZM-bl NAb Assay in a GCLP-compliant environment, endorsed laboratories are now in a position to function as regional centers to conduct the assay for current and future clinical trials. Additionally, the endorsed laboratories are now serving as training centers for both assay related tasks and for GCLP compliance. As the popularity of the TZM-bl NAb Assay spreads, it is crucial that a structure of regional testing and training centers exist so that clinical trial research can be conducted in the areas where the clinical trials occur [11], [12], [18]. To date, five of the laboratories have already or will soon test samples for Phase I and/or II clinical trials in their region. Additionally, the laboratories can also use their expertise in the TZM-bl NAb Assay and GCLP to solicit and procure additional sources of funding for future projects. This becomes important as more research sponsors are mandating laboratory compliance to national/international standards and regulations as a contingency for funding. This program also serves as a model to implement newer HIV neutralization assays. Within the CA-VIMC, efforts are already underway to transfer the new Neutralizing Antibody Assay for HIV-1 in A3R5 Cells to its laboratories. In addition, this technology transfer process could serve as a guideline for transferring other standardized assay technologies to laboratories worldwide.
